# Mediterranean Dietary Treatment in Hyperlipidemic Children: Should It Be an Option?

**DOI:** 10.3390/nu14071344

**Published:** 2022-03-23

**Authors:** Giulia Massini, Nicolò Capra, Raffaele Buganza, Anna Nyffenegger, Luisa de Sanctis, Ornella Guardamagna

**Affiliations:** 1 Department of Public Health and Pediatric Sciences, University of Turin, 10126 Turin, Italy; raffaele.buganza@unito.it (R.B.); annanyffe@gmail.com (A.N.); luisa.desanctis@unito.it (L.d.S.); 2 Pediatric Endocrinology Unit, Regina Margherita Children’s Hospital, 10126 Turin, Italy; 3 Centro Cardiologico Monzino, IRCCS, 20138 Milan, Italy; nicolo.capra@cardiologicomonzino.it

**Keywords:** primary hyperlipidemia, dietary treatment, children, Familial Hypercholesterolemia, polygenic hypercholesterolemia, Mediterranean diet, LDL-C therapeutical target

## Abstract

Background: Diet is considered the cornerstone of lipid management in hyperlipidemic children but evidence to demonstrate the effects of nutrient benefits on the lipid profile is limited. Aim: The aim of this study is to evaluate the impact of the Mediterranean diet on low-density lipoprotein (LDL-C) and non-high density lipoprotein (HDL-C) decrease in primary hyperlipidemia affected children and in the achievement of therapeutical target levels. Methods: A retrospective cohort study was used, recruiting *n* = 223 children (10.05 ± 3.26 mean age years) with familial hypercholesterolemia (FH) (*n* = 61, 27%) and polygenic hypercholesterolemia (PH) (*n* =162, 73%). Secondary hyperlipidemias were excluded. Based on LDL-C and non-HDL-C decrease, participants were divided into two groups, named the Responder Group and Non-Responder Group. Participants and their families underwent dietary education by an expert nutritionist and were asked to fill in a weekly diary to be delivered at visits. Dietary indications were in line with daily caloric requirement, daily food quality and quantity intakes typical of the Mediterranean diet. These include carbohydrates, extra virgin olive oil, yoghurt and milk derivatives, fish and vegetable proteins, fresh seasonal vegetables and fresh fruits. Nuts or almonds were also recommended. The advice to limit intakes of meat, in particular red meat, and caution against junk food and sugar added food and beverages was provided. At medical visits, carried out at baseline (T0) and 6 months later (T1), children underwent anthropometric measurements and blood collection. Standard kits and methods were applied for lipid analysis. Statistical methods were performed by SAS version 9.4 (SAS Institute, Cary, NC, USA). Signed informed consent was given by parents according to the Declaration of Helsinki and the study was approved by the Local Committee. Results: The Responder Group (*n* = 156/223, 70%) included 45 FH and 111 PH children, while the Non-Responder Group (*n* = 67/223, 30%) included 16 FH and 51 PH children. The Responder Group showed total cholesterol (TC), LDL-C and non-HDL-C median percentage decreases of 9.45, 13.51 and 10.90, respectively. These statistically significant changes (*p* ≤ 0.0001) were similar in the FH and PH subgroups but just PH subjects reached the LDL-C and non-HDL-C target, which fell below 130 mg/dL and 145 mg/dL, respectively. Saturated fatty acids (SFAs) were the main dietary parameter that distinguished between the Responder Group and the Non-Responder Group (*p* = 0.014). Positive correlations were found at T1 between dietary total lipids, SFAs and cholesterol with serum LDL-C, non-HDL-C and TC variations. These latter serum parameters had an inverse correlation with dietary carbohydrate at T1. Among macronutrients, SFAs were finally demonstrated to be the predictor of serum lipids variation at T1. Conclusions: The dietary intervention with a Mediterranean diet in children with primary hyperlipidemia significantly improves the lipid profile both in FH and PH subgroups and allows target levels of LDL-C and non-HDL-C in PH subjects to be reached. Responsiveness benefits should be primarily attributed to the reduction in SFAs, but changes in dietary lipids, cholesterol and carbohydrate intake may also play a role. In contrast, the Non-Responder Group showed a worsening of lipid profile regarding the unchanged diet.

## 1. Introduction

The prevention of atherosclerosis begins early in life; therefore, cardiovascular (CV) risk factors, as a burden of disease, have a strong impact on health outcomes in adulthood [1]. Among risk factors, hyperlipidemia plays a huge role in pediatrics, thus, an early and final diagnosis is of great importance to prevent CV damage. In these conditions, the first line of a therapeutical approach is represented by a healthy lifestyle and nutrition.

The effectiveness of reducing total fat and cholesterol intakes to improve low-density lipoprotein cholesterol (LDL-C) serum levels has been demonstrated [2]. This approach has been shown to be safe in children when growth and pubertal development were considered; it is therefore extensible to the entire population and to children suffering primary dyslipidemia [3]. Furthermore, the intake of saturated fatty acids (SFAs) was positively associated with serum total cholesterol (TC), LDL-C and Apolipoprotein B (ApoB) levels in Familial Hypercholesterolemia (FH) affected children [4]. Although the role of SFAs is still discussed [5], the replacement of SFAs with polyunsaturated fatty acids (PUFAs), as demonstrated by randomized clinical trials and meta-analysis, reduced the risk of CV events by 27% [6] and serum TC by 15–30% [4]. In FH children, interventions that modify dietary fat [7,8], in addition to protein [9] or fiber intakes [10], are still discussed, while more attention is currently being given to healthy food than to a single macronutrient [6,11].

In high-risk children, such as those affected by FH, the LDL-C target level is considered safe when ≤130 mg/dL (3.5 mmol/l) [12], while a further decrease to LDL-C ≤ 110 mg/dL (2.84 mmol/L), corresponding to around the 90th percentile, could improve the benefits [13,14].

The aim of the present study was to evaluate the efficacy of the Mediterranean diet on serum lipid profile amelioration in children affected by primary hypercholesterolemia, including FH and polygenic hypercholesterolemia (PH). A significant decrease in LDL-C and non-HDL-C were the primary end points, while the secondary end point was the achievement of therapeutical target levels.

## 2. Methods

### 2.1. Study Design

A retrospective cohort study was carried out on hyperlipidemic children referred to the Outpatients Lipid Clinic of the Regina Margherita Children’s Hospital of Turin in the period 2016–2019. From the analysis of the outpatient database, children were screened on basis of the eligibility criteria as indicated below. Enrolled children included FH and PH subjects. At time of diagnosis, and at least 6 months before the beginning of the present study, they had already been instructed to switch from free diet to CHILD I then to CHILD II diets [14]. The recruited participants underwent two visits, at baseline (T0) and 6 months later at follow-up (T1). On these occasions they were submitted to clinical and auxological evaluation, serum lipid levels analysis and dietary record control. All participants were assigned to the Mediterranean diet style at T0. Serum lipids were evaluated on 12 h fasting blood sample and laboratory tests were processed by an automated method (Integra plus 400/Cobas 6000) with commercial kits (automated Modular P3 analyzer- Roche). The LDL-C value was calculated according to the Friedewald equation: TC-HDL-C-TG/5 [15]; non-HDL-C was obtained from the difference between TC and HDL-C. Participants were grouped into the Responder (R) Group or the Non-Responder (NR) Group based on serum LDL-C and non-HDL-C decrease at T1. The study was conducted according to the Helsinki declaration and was approved by the local Ethical Committee. Written informed consent was obtained from the participants and the parents.

### 2.2. Participants

**Figure 1:**
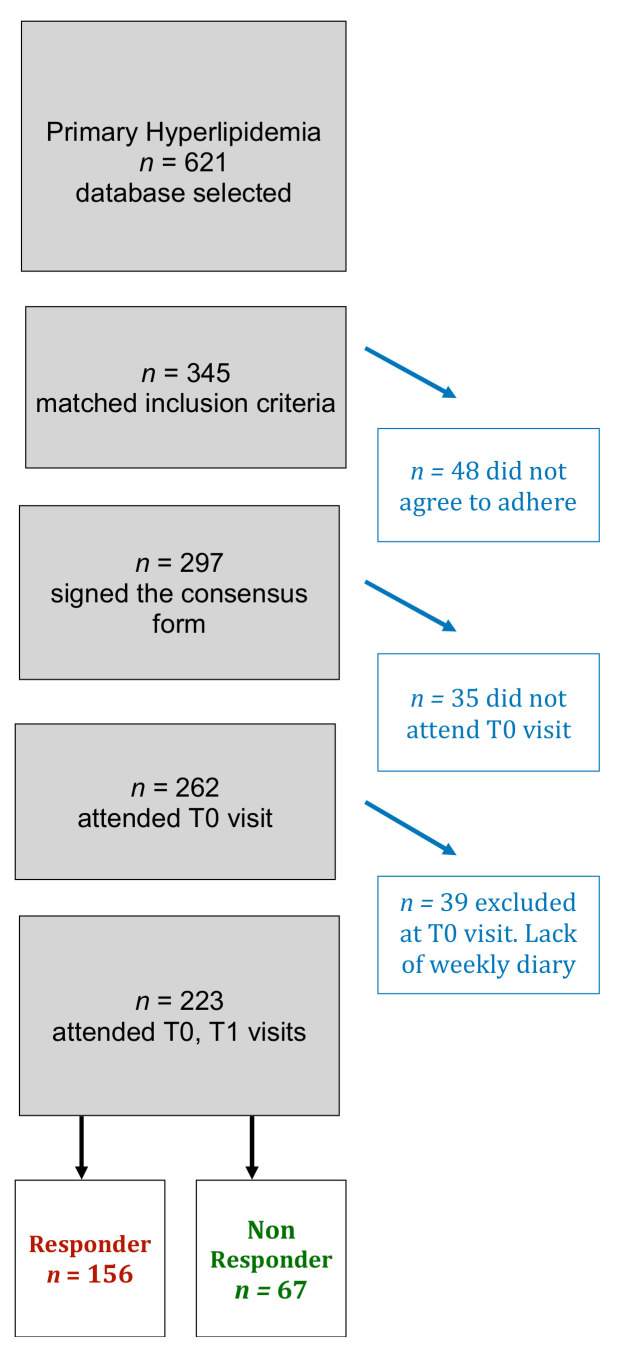
Flow-chart of participants to the study.

The number of eligible children was 223, 10.05 ± 3.26 mean age years, affected by FH (*n* = 61, 27%) or PH (*n* = 162, 73%) (Figure 1). Children were clinically healthy at time of diagnosis of primary hyperlipidemia, based on a selective familial screening, and were prepubertal. They were asked not to change their physical activity between time T0 and T1. The family tree was examined for two generations to detect the disorder inheritance. The diagnosis of FH was made according to the following criteria: LDL-cholesterol levels ≥ 95th percentile (age, sex-specific); dominant inherited hypercholesterolemia; family history of precocious CV event in a parent or grandparent; LDL-R gene mutation analysis [16]. Children were diagnosed as PH when serum LDL-C exceeded the 90th percentile but they did not fulfill the above criteria for inclusion in the FH subgroup. Secondary forms of dyslipidemia were excluded, i.e., renal disease, liver disease, hyperthyroidism or hypothyroidism, overweight and obesity, hyperglycemia or diabetes, immune-hematological disorders. Patients undergoing phytosterols or hypolipidemic drugs in the last three months were excluded as well as subjects without exhaustive food week diaries at baseline T0 and/or T1.

### 2.3. Diet

During the first visit at T0 the participants and their parents received education and dietary guidelines, according to the Mediterranean diet style, from an expert nutritionist in a face to face meeting. The recommended dietary pattern was characterized by a wide consumption of cereals (3–4 times/day), legumes (2–3 times/week), fish (2–3 times/week), plant foods (vegetables and fruit 4 times/day), nuts (20–30 g/day), extra virgin olive oil as the main source of fat (3–4 spoon /day), moderate consumption of fresh dairy products (2 times/day) and eggs (2–3/week), small amount of meat, especially red meat (1/week). They were instructed to limit sweets (allowed just 1/week), to avoid drinks with added sugar, to exclude processed food, and meat in particular, margarine, snacks, or junk food. Furthermore, they were asked to eat respecting schedules and to observe three main meals, which included breakfast, lunch and dinner. Participants and their families were given a weekly diary form they were asked to fill in during the week preceding visits at time T0 and T1 when the diary was to be delivered. The form included 12 food classes (cereals, fish, meat, dairy, legumes, vegetables, fruit, nuts, fats, pastries/sweets, beverages, snacks) and 36 common items that indicated, for any food, the weight consumed at each meal, or referred to as tablespoon, teaspoon, cup or bowl. Participants were asked to include any other food not listed in the diary form and they were also shown a booklet with pictures of the most common food portions [17]. During the visits, the diary report was discussed with the nutritionist to verify the quality, accuracy, compliance to dietary indications, Mediterranean style-related, to ascertain any shortcomings or doubts to be evaluated in detail. This latter procedure was preliminary to examine the food quantity: the seven-days weight of each food category were summarized, each macronutrient estimated over a weekly period and an average daily food intake classified in grams and percentage of Energy (E). The quantitative assessment of macronutrients was estimated to calculate the intake of energy and nutrients, according to INRAN and BDA food composition databases [18,19]. The total calories recommended to enrolled patients were calculated evaluating basal metabolism (Schofield equation), which considers age, sex, weight and physical activity level, classifying children into inactive, moderately active or active. All dietary indications related to micronutrients were respected to pursue children’s correct growth, according to the Reference Intake Levels for the Italian Population (LARN). Intakes of nutrients and energy were then matched and evaluated in a combined manner. Weekly food diaries were therefore analyzed to compare food intakes at T0 and T1. Participants received a phone call every two months to update, support doubts and to verify adherence to the nutritional indications.

### 2.4. Statistical Analysis

Continuous variables were expressed as mean ± standard deviation (SD), skewed variables as median (25th–75th percentile), while categorical variables were shown as absolute numbers and percentages. The Chi-square test or Fisher’s exact test were performed for analysis that involved categorical variables. Unpaired t-test or Kruskal–Wallis test were performed to compare lipids and nutrients intake between two groups (Responder vs. Non-Responder). A paired T-test or a Wilcoxon signed-rank test was applied to evaluate differences for these variables between the two measurements, within the two groups. Partial correlation (adjusted for age and sex) was performed using Pearson’s test to evaluate the association between LDL-C, non-HDL-C and TC variations with different macronutrients. The variables found to be significantly associated with the outcomes (∆ LDL-C, ∆ non-HDL-C, ∆ TC) were involved in a linear regression stepwise. The association between macronutrient levels at T1 and the responsiveness status was assessed by logistic regression. Odds ratio adjusted for age and sex and 95% confidence limits were reported. All skewed variables involved were log-transformed. A sub-analysis was conducted to evaluate differences within the R-Group between patients FH and PH affected. All tests were 2-tailed, and a *p* ≤ 0.05 was considered statistically significant. All analyses were performed using SAS version 9.4 (SAS Institute, Cary, NC, USA).

## 3. Results

The baseline characteristics of the study cohort are reported in Table 1. All 223 recruited participants completed the study as compliant after filling in the weekly diaries. The R-Group (*n* = 156, 70%) included 45 FH (29%) and 111 PH (71%) subjects, while the NR-Group (*n* = 67, 30%) included 16 FH (24%) and 51 (76%) PH subjects. The R-Group showed median basal serum TC levels of 226 mg/dL (206–253), LDL-C levels of 148 mg/dL (128–175) and non-HDL-C levels of 166 mg/dL (144–192). These values were higher when compared to the NR-Group, which showed median serum TC levels of 214 mg/dL (187–239), LDL-C levels of 133 mg/dL (114–162) and non-HDL-C levels of 153 mg/dL (135–186). The FH subgroup included 73.8% R and 26.2% NR subjects, while in the PH subgroup 68.5% were R and 31.5% NR.

The decrease in serum lipids in the R-Group, comparing T0 and T1, also included a decrease in LDL-C and non-HDL-C with percentage drops of 9.45, 13.51 and 10.90, respectively. Each of these changes was statistically significant (*p* ≤ 0.0001) (Table 2).

At the same time, the nutrient variations in the R-Group showed a significant decrease in total dietary lipids (T0 33.8% Energy(E) vs. T1 32.8 % E; *p* = 0.0329), dietary cholesterol (T0 137 mg/day vs. T1 125 mg/day; *p* = 0.0471) and SFAs (T0 10%E vs. T1 8.8%E; *p* ≤ 0.0001) intakes. Furthermore, the intake of carbohydrates (T0 50.3% E vs. T1 51.3% E; *p* = 0.0430) and fiber (10 g/day vs. 11.37 g/day; *p* ≤ 0.0001) increased. Conversely, the NR-Group did not show any significant difference as regards to nutritional parameters. SFAs were the only dietary parameter distinguishing the R and the NR groups (*p* = 0.014) when considering T1–T0 differences, while a trend towards decreasing total lipid levels was observed (*p* = 0.059) (Table 2).

Both FH and PH subgroups of the R-Group ameliorated lipid parameters from T0 to T1 with no variations between the two subgroups. On the contrary, a statistically significant change in each subgroup was detected. Both FH and PH demonstrated a decrease in TC, from 272 mg/dL to 245 mg/dL (*p* ≤ 0.0001) and from 216 mg/dL to 192 mg/dL (*p* ≤ 0.0001), respectively; LDL-C from 205 mg/dL to 177 (*p* ≤ 0.0001) and from 137 mg/dL to 117 mg/dL (*p* ≤ 0.0001), respectively; non-HDL-C from 222 mg/dL to 190 mg/dL (*p* ≤ 0.0001) and from 153 mg/dL to 133 mg/dL (*p* ≤ 0.0001), respectively (Table 3). Furthermore, the corresponding dietary intakes are the same as those already described in the R-Group.

**Figure 2:**
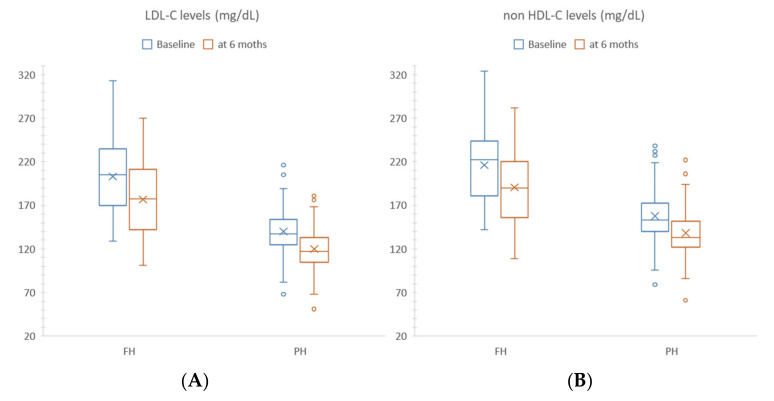
LDL-C (**A**), non-HDL-C (**B**) Box-plot at baseline (blue) and at 6 months follow-up (orange) in familial hypercholesterolemia (FH) and polygenic hypercholesterolemia (PH) in the Responder Group.

The PH subgroup reached the targets of LDL-C ≤ 130 mg/dL and non-HDL-C ≤ 145 mg/dL, while FH children maintained serum LDL-C values above the 95th percentile (Figure 2).

Considering the three dependent variables LDL-C, non-HDL-C and TC, a positive correlation with total lipids and SFAs and a negative correlation with carbohydrates was observed at T1. For the non-HDL-C variation, dietary cholesterol was also positively associated (Table 4).

**Figure 3:**
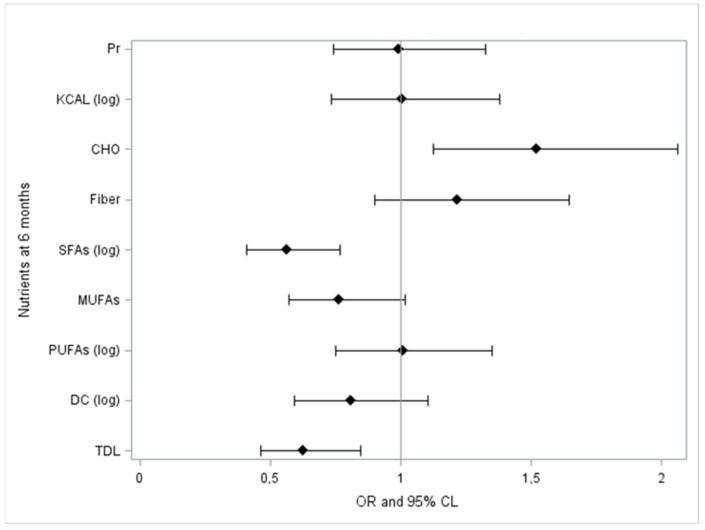
Standardized odds ratio of macronutrients at T1 adjusted for age and sex. CL, 95% confidence limits.

The logistic regression analysis underlines significant associations (Figure 3). These include dietary total lipids (OR 0.626; 95% CL 0.463–0.847; *p*-value 0.002) and SFAs (OR 0.561; 95% CL 0.41–0.768; *p*-value 0.0003) that are negatively associated to serum lipids improvement at T1, while carbohydrates (OR 1.522; 95% CL 1.124–2.061; *p*-value 0.007) show a favorable impact on this outcome.

To detect which parameter might play a predictive role, the stepwise linear regression was used, showing SFAs as the most important and unique predictor of serum lipids variation (Table 5). Furthermore, SFAs are also related to the responsive outcome (also adjusting for carbohydrate and total lipids intakes at T1, as well as for sex and age with OR: 0.598 (0.390; 0.919), data not shown).

## 4. Discussion

The present study is intended to ascertain the effects of the Mediterranean dietary intervention on the lipid profile in children undergoing a diagnosis of primary hyperlipemia. The recruited cohort included FH and PH children showing an increased CV risk related to their diagnosis, which was familial inherited, and requiring early detection to optimize the treatment. The efficacy of the dietary intervention is confirmed by the decrease in the LDL-C and non-HDL-C median values observed in the R-Group. In this group, the median LDL-C value dropped below 130 mg/dL at the same time as a dietary intake reduction in total lipids, cholesterol and SFAs, as well as an increased consumption of carbohydrate and fiber. Serum lipid parameters demonstrate a positive relationship with dietary macronutrients: correlations between serum LDL-C, non-HDL-C and TC variations with dietary lipids, SFAs and cholesterol intake were detected six months after the shift to the Mediterranean dietary style. Similar considerations apply to the FH and PH subgroups, whose changes reflect those observed in the whole R-Group.

The SFA parameter results show, overall, the main and most consistent variable distinguishing the R-Group from the NR-Group. SFAs are finally confirmed as the main predictive marker of LDL-C, non-HDL-C and TC variables six months after dietary changes. Our results are concordant with Giannini et al.’s conclusions: they found a 10% LDL-C drop after 6–12 months of a Mediterranean diet in hypercholesterolemic children, although they were not affected by primary inherited disorders [20]. Conversely, another study, which also considered hyperlipidemic children on a Mediterranean diet, found no correlations between the KIDMED score and serum lipids [21]. The efficacy of the dietary treatment for primary hyperlipidemias, particularly for FH subjects, is still being discussed [6,22,23]. Anyway, the dietary SFAs’ variable is more commonly recognized as being positively related to serum LDL-C concentrations, and there is consensus on its benefits to attain safe LDL-C levels, as shared by International Guidelines [12,14,16].

LDL-C and non-HDL-C levels are the main recognized targets for CV prevention, thus marking the path to follow. The PH subgroup reached the established safety goal as mean LDL-C and non-HDL-C values did not exceed 130 mg/dL and 145 mg/dL at T1, respectively, thus reaching our primary target [24,25]. This finding underlines the effectiveness of the current program in this cohort.

FH children benefited as well from this approach and reached the primary aim point, but not all cases achieved the therapeutical target objective. The elevated baseline values and the great phenotypic heterogeneity within this group may explain this observation. Further dietary restrictions and/or the administration of supplementary foods should likewise improve the serum lipid level decrease before moving to drugs. This approach has been largely and successfully demonstrated by sterol/stanol dairy supply [12,26]. Any therapeutical final decision aimed at reaching the LDL-C goal needs to be considered deeply. FH children frequently require pharmacological intervention, but once again, diet is confirmed as the first mandatory step to be considered and followed life-long [27]. Educational and nutritional interventions from childhood are have a relevant impact on eating habits in adulthood [28].

Dietary intervention measures improved the serum lipid profile in 70% of participants, mirroring the similar percentage distribution of FH and PH subjects, as well as that of the adherence to the dietary advice. Conversely, for subjects belonging to the NR-Group their lipid profile was unchanged or was worse (30%). This outcome can be related to the great nutritional differences observed within and between the R-Group and the NR-Group; the effect underlining the impact of paying attention to the advice. The failure to improve the lipid profile in the NR-Group might be partly ascribed to the challenging seven-day program that participants were asked to adhere to, which required subjects to mark and specify any quality and quantity of a consumed food a demanding request. Moreover, changing eating habits is a challenging task; it has many implications that limit its application, although the awareness of healthy nutrition is recognized greater in FH subjects than in non-FH subjects [28]. It should also be mentioned that participant’s baseline nutritional data were partially acceptable as they had been previously educated to attain indications in line with the Expert Panel Guidelines [14].

Lifestyle measures represent the first line therapeutic approach to prevent CV outcome disorders; thus, the American Academy of Pediatrics’ guidelines indicate the macronutrient daily intakes as potentially effective [12,14]. It is questionable whether the need to reduce high LDL-C levels, as frequently observed in FH children, indicates drugs to be applied as the urgent and final solution [12,26]. The Mediterranean diet, alternatively, has been demonstrated to be effective in improving the lipid profile and to reduce CV events and the risk of chronic degenerative disorders in adults [29,30], while most papers addressed the focus on children and overweight/obesity, allergies, and respiratory disorders [31]. Scant data exist on lipoprotein disorders [20] and no data link this eating habit to primary hyperlipidemia in children; thus, the Mediterranean diet has not been considered in pediatric guidelines at present. These two dietary approaches have been successfully applied in different countries: the Expert Panel guidelines, typical of Western countries, addresses the defined quantitative macronutrient intakes, while the Mediterranean dietary style emphasizes the assumption of healthy foods typical of Mediterranean areas, with low nutrient calories, and suggests only serving a number of proposals [32]. Anyway, both nutritional models share common features as characterized by the low content of saturated fats and cholesterol, the elevated level of monounsaturated fats, the higher intake of calories with carbohydrates and the quite similar content of proteins. The hallmarks of the Mediterranean diet style can be summarized as “food as a whole and first,” while for the Expert Panel diet is “mathematic diet evidence based.” Both have been shown to be effective in reaching a decrease in LDL-C [11]. We observed the beneficial effects in the R-Group, although the dietary mean total fat intake exceeded 30% of the daily energy, with the reduction in SFAs in favor of unsaturated fatty acids and a cholesterol intake below 150 mg/day [6,33]. This contrasts with the guidance of the Expert Panel but agrees with Mediterranean dietary style reports [29,34].

Adequate dietary intervention requires a deep education of the whole family, although they will already be aware of the negative effects of hyperlipidemia on CV health. This has implications for nutritional education and daily habits. Education regarding the nutritional benefits or limitations of any food, and the appropriate intake, the observance of eating style and the respect for the daily schedule are relevant in order to achieve the purpose. The prerequisite to improve compliance with feeding recommendations is represented by the team including a physician, nutritionist and nurse who can encourage families to reach the goal. Food is a mixture of components each with different effects, so it is important to underline the quality, as well as servings, and to progressively induce the required changes. The different ethnic culture should not be underestimated since it has been demonstrated that benefits can be reached by a different nutritional approach [11].

The familial socioeconomic level, and the subjects physical activity and behavioral status further contribute to modify serum lipid levels, but in this retrospective study, exhaustive data were not fully available and the present results are not adjusted for these variables. The impact of these parameters on serum lipid change, and also the pubertal stage, needs to be considered in further extensive studies to reach an exhaustive understanding of the effect of the Mediterranean diet, not only in children but in adolescents as well.

The responsiveness to this program was closely related to the adherence to the Mediterranean diet, both reached by two-thirds of participants. This observation has implications in clinical practice as it prompts the question as to the best way to pursue and evaluate nutritional intake and encourage compliance. The adherence to the Mediterranean diet is commonly estimated by different scores, adapted to different ages [35,36]. In the present study, we did not apply any score but we tested the compliance and efficacy, combining the food quality levels and quantity intakes in a weekly period that was representative of children’s usual habits. Adherence has been commonly achieved by families of Mediterranean origin, although children would move to a Western eating style, as already reported [37].

The strengths of the study consist of several points: First, the selected participants did not receive drugs or supplementary foods and were selected from a narrow age range, to avoid any bias referable to adolescence lipid variation. Second, the compilation of the weekly food diary, the longest period ever applied to date, reflects the more strict habitual food intake and likely reduces misclassification. Third, the temporal correspondence between serum lipid evaluation and the completion of the weekly diary. Fourth, the deep nutritional assessment, which combines both qualitative, according to the Mediterranean diet, and quantitative food intake, as indicated by AHA guidelines.

Limitations have also to be recognized. First, the investigated case series consists of a limited number of participants and second, the diary self-reported data could under/overestimate the amount of food or contain mistakes. Third, the absence of a control group, although the NR-Group diary reports support the poor adherence to dietary instructions and are associated with unimproved serum lipid levels. On this basis, it seems to be acceptable to compare the R-Group with the NR-Group and to support the above findings.

## 5. Conclusions

The Mediterranean diet is of benefit to primary hyperlipidemia affected children who reached the established primary end point as both FH and PH participants significantly reduced serum LDL-C and non-HDL-C levels. The secondary end point of lowering LDL-C below 130 mg/dL and non-HDL-C below 145 mg/dL was achieved by PH subjects. The nutritional lifestyle was suitable to reduce SFAs, the main parameter here confirmed, once again, as predictive of serum lipid changes. However, if diet represents the first line treatment requiring great attention, it should be underlined that FH children need strict monitoring to add supplementary foods and to switch to hypolipidemic drugs, once required, as the detrimental effect of increased levels of LDL-C on the clinical outcomes in adulthood has been demonstrated.

## Figures and Tables

**Table 1:** Baseline characteristics of the study population.

Parameter	Unit	ALL (n°223)	NR (n°67)	R (n°156)	*p*-Value
AGE	year	10.05 ± 3.26	10.18 ± 3.45	9.99 ± 3.19	0.699
SEX (MALE)		101 (45.3%)	27 (40.3%)	74 (47.7%)	0.326
BMI	kg/m^2^	18.84 (16.26; 22.05)	19.5 (16.9; 23.49)	18.65 (16.19; 21.6)	0.158
TC	mg/dL	220 (202; 251)	214 (187; 239)	226 (206; 253)	0.004 *
HDL-C	mg/dL	58 (49; 68)	57 (47; 68)	59 (50; 68)	0.141
LDL-C	mg/dL	145 (124; 174)	133 (114; 162)	148.5 (128.5; 175)	0.003 *
TG	mg/dL	69 (55; 104)	74 (59; 112)	67.5 (54; 99)	0.051
Non-HDL-C	mg/dL	159 (141; 189)	153 (135; 186)	166 (144.5; 192.5)	0.017 *
Calories	Kcal/day	1258 (10,472; 1441)	1236 (1056; 1441)	1259.5 (1084; 143)	0.649
TDL	E%/day	34.06 ± 5.01	34.63 ± 5.67	33.82 ± 4.69	0.273
DC	mg/day	137.7 (107.77; 174.31)	136 (106; 177.85)	137.85 (109.32; 172.99)	0.812
PUFAs	E%/day	3.3 (2.9; 3.81)	3.35 (2.81; 3.95)	3.29 (2.93; 3.8)	0.735
MUFAs	E%/day	16.36 ± 3.45	16.86 ± 3.98	16.14 ± 3.19	0.153
SFAs	E%/day	10.15 (8.44; 11.63)	10.16 (8.68; 11.41)	10.08 (8.2; 11.7)	0.706
Fiber	g/day	9.96 ± 3.58	9.64 ± 3.95	10.09 ± 3.41	0.387
CHO	E%/day	50.05 ± 5.82	49.56 ± 5.94	50.26 ± 5.78	0.412
Pr	E%/day	15.83 ± 2.65	15.78 ± 2.27	15.86 ± 2.81	0.837

NR, non-responders; R, responders; BMI, body mass index; TC, total cholesterol; HDL-C, high-density lipoprotein cholesterol; LDL-C, low-density lipoprotein cholesterol; TG, triglycerides; TDL, total dietary lipids; DC, dietary cholesterol; PUFAs, polyunsaturated fatty acids; MUFAs, monounsaturated fatty acid; SFAs, saturated fatty acids; CHO, carbohydrates; kcal, kilocalories; Pr, proteins; E%/day, daily percentage of energy intake; *, statistically significant *p*-value ≤ 0.05.

**Table 2:** Lipid profile and daily dietary intake at baseline (T0) and at 6 months follow-up (T1) and estimated changes (Delta) between the Responder Group and the Non-Responder Group.

Parameter	Unit	NON-RESPONDERS (n°67)			RESPONDERS (n°156)			
		T_0_	T_1_	*p*-value	T_0_	T_1_	*p*-value	∆ *p*-value
AGE	year	10.18 ± 3.45		-	9.99 ± 3.19		0.699	
SEX (MALE)		27 (40.3%)		-	74 (47.7%)		0.326	
BMI	kg/m^2^	19.5 (16.9; 23.49)	20.08 (16.11; 23.11)	0.097	18.65 (16.19; 21.66)	18.87 (16.2; 21.44)	0.826	0.159
TC	mg/dL	214 (187; 239)	222 (200; 245)	<0.0001 *	226 (206; 253)	202.5 (181; 233)	<0.0001 *	<0.0001
HDL-C	mg/dL	57 (47; 68)	57 (48; 64)	0.978	59 (50; 68)	55 (47; 65)	<0.0001 *	0.016 *
LDL-C	mg/dL	133 (114; 162)	148 (123; 179)	<0.0001 *	148.5 (128.5; 175.5)	127 (109.5; 156)	<0.0001 *	<0.0001
TG	mg/dL	74 (59; 112)	83 (61; 99)	0.241	67.5 (54; 99)	71 (52; 97)	0.787	0.483
Non-HDL-C	mg/dL	153 (135; 186)	164 (140; 191)	<0.0001 *	166 (144.5; 192.5)	144.5 (125; 176.5)	<0.0001 *	<0.0001
Calories	Kcal/day	1236 (1056; 1441)	1208 (1104; 1368)	0.745	1259.5 (1084; 1436)	1254.6 (1083; 1383.5)	0.503	0.644
TDL	E%/day	34.63 ± 5.67	35.45 ± 6.15	0.352	33.82 ± 4.69	32.84 ± 5.02	0.033 *	0.059
DC	mg/day	136 (106; 177.85)	133 (105; 160)	0.419	137.85 (109.32; 172.99)	125.15 (99.5; 168.33)	0.047 *	0.321
PUFAs	E%/day	3.35 (2.81; 3.95)	3.4 (3.02; 3.92)	0.444	3.29 (2.93; 3.8)	3.33 (2.9; 3.87)	0.362	0.736
MUFAs	E%/day	16.86 ± 3.98	17.42 ± 3.79	0.361	16.14 ± 3.19	16.31 ± 3.76	0.628	0.619
SFAs	E%/day	10.16 (8.68; 11.41)	10.5 (8.8; 12.38)	0.51	10.08 (8.2; 11.7)	8.84 (7.61; 10.48)	<0.0001 *	0.014
Fiber	g/day	9.64 ± 3.95	10.62 ± 3.91	0.057	10.09 ± 3.41	11.37 ± 3.89	<0.0001 *	0.642
CHO	E%/day	49.56 ± 5.94	48.71 ± 6.58	0.375	50.26 ± 5.78	51.31 ± 5.77	0.043 *	0.077
Pr	E%/day	15.78 ± 2.27	15.87 ± 2.12	0.797	15.86 ± 2.81	15.82 ± 2.49	0.895	0.821

BMI, body mass index; TC, total cholesterol; HDL-C, high-density lipoprotein cholesterol; LDL-C, low-density lipoprotein cholesterol; TG, triglycerides; TDL, total dietary lipids; DC, dietary cholesterol; PUFAs, polyunsaturated fatty acids; MUFAs, monounsaturated fatty acid; SFAs, saturated fatty acids,: CHO, carbohydrates; kcal, kilocalories; Pr, proteins; *p*-value: T1–T0 differences in each group; ∆ *p*-value: comparison of T1–T0 differences between R and NR; E%/day, daily percentage of energy intake; *, statistically significant *p*-value ≤ 0.05. ∆ *p*-values are sex and age adjusted. For age and sex, the *p*-value was reported regarding difference at baseline between the two groups.

**Table 3:** Lipid profile and daily dietary intake at baseline (T0) and at 6 months follow-up (T1) and estimated changes (Delta) between the FH and PH in the Responder Group.

Parameter	Unit	FH RESPONDERS (n°45)			PH RESPONDERS (n°111)			
		T_0_	T_1_	*p*-value	T_0_	T_1_	*p*-value	∆ *p*-value
AGE	year	9.37 ± 3.52		-	10.24 ± 3.03		0.125	
SEX (MALE)		19 (42.2%)		-	55 (49.55%)		0.406	
BMI	kg/m^2^	18.18 (15.04; 19.73)	17.64 (15.68; 19.93)	0.946	18.9 (16.3; 22.32)	19.19 (16.43; 22.06)	0.802	0.942
TC	mg/dL	272 (234; 306)	245 (214; 276)	<0.0001 *	216 (198; 237)	192 (178; 216)	<0.0001 *	0.388
HDL-C	mg/dL	55 (48; 64)	54 (45; 61)	0.581	61 (51; 72)	55 (47; 67)	<0.0001 *	0.077
LDL-C	mg/dL	205 (170; 235)	177 (142; 211)	<0.0001 *	137 (125; 155)	117 (105; 133)	<0.0001 *	0.380
TG	mg/dL	63 (53; 75)	63 (44; 84)	0.940	68 (55; 107)	73 (53; 106)	0.871	0.779
Non-HDL-C	mg/dL	222 (181; 244)	190 (156; 220)	<0.0001 *	153 (140; 173)	133 (122; 153)	<0.0001 *	0.806
Calories	Kcal/day	1308 (1122; 1453)	1300 (1116; 1488)	0.993	1232 (1083; 1430)	1243 (1070; 1372)	0.409	0.723
TDL	E%/day	33.31 ± 4.88	32.72 ± 5.87	0.530	34.03 ± 4.62	32.89 ± 4.67	0.030 *	0.488
DC	mg/day	142.14 (104.48; 175. 5)	127.24 (95; 172)	0.438	137.7 (112; 171.67)	123.12 (100.3; 159)	0.057	0.858
PUFAs	E%/day	3.36 (3.06; 3.81)	3.19 (2.72; 3.81)	0.656	3.25 (2.87; 3.8)	3.4 (2.94; 3.9)	0.097	0.079
MUFAs	E%/day	16.34 ± 3.22	16.44 ± 4.31	0.889	16.06 ± 3.18	16.25 ± 3.54	0.946	0.937
SFAs	E%/day	9.48 (7.5; 11.12)	7.99 (6.98; 10.55)	0.040 *	10.2 (8.44; 11.85)	9.11 (7.8; 10.46)	0.002 *	0.898
Fiber	g/day	10.26 ± 3.39	11.51 ± 3.17	0.022 *	10.03 ± 3.42	11.31 ± 4.16	0.001 *	0.929
CHO	E%/day	51.26 ± 5.82	5.96 ± 5.53	0.503	49.85 ± 5.74	51.05 ± 5.86	0.047 *	0.566
Pr	E%/day	15.38 ± 2.64	15.36 ± 2.4	0.950	16.05 ± 2.86	16.01 ± 2.51	0.908	0.933

FH, familial hypercholesterolemia; PH, polygenic hypercholesterolemia; BMI, body mass index; TC, total cholesterol; HDL-C, high-density lipoprotein cholesterol; LDL-C, low-density lipoprotein cholesterol; TG, triglycerides; TDL, total dietary lipids; DC, dietary cholesterol; PUFAs, polyunsaturated fatty acids; MUFAs, monounsaturated fatty acid; SFAs, saturated fatty acids; CHO, carbohydrates; kcal, kilocalories; Pr, proteins; *p*-value: T1–T0 differences in each group; ∆ *p*-value: comparison of T1–T0 differences between FH and PH; E%/day, daily percentage of energy intake; *, statistically significant *p*-value ≤ 0.05. ∆ *p*-values are sex and age adjusted. For age and sex, the *p*-value was reported regarding difference at baseline between the two groups.

**Table 4:** Pearson’s partial correlation index between macronutrients at T1 and serum lipid variation, sex and age adjusted.

Time T1	Delta Log LDL-C	Delta Log Non-HDL-C	Delta Log TC
TDL	0.152	0.172	0.137
	0.024 *	0.010 *	0.041 *
DC (log)	0.128	0.132	0.106
	0.057	0.0499 *	0.115
PUFAs (log)	−0.012	0.018	0.007
	0.854	0.789	0.919
MUFAs	0.098	0.104	0.063
	0.147	0.124	0.348
SFAs (log)	0.186	0.224	0.204
	0.006 *	0.001 *	0.002 *
F	−0.056	−0.085	−0.065
	0.4064	0.2059	0.3341
CHO	−0.168	−0.201	−0.152
	0.0124 *	0.003 *	0.024 *
KCAL (log)	0.032	0.002	0.043
	0.635	0.976	0.521
Pr	0.080	0.111	0.072
	0.238	0.101	0.285

TDL, total dietary lipids; DC, dietary cholesterol; PUFAs, polyunsaturated fatty acids; MUFAs, monounsaturated fatty acid; SFAs, saturated fatty acids; F, fibers; CHO, carbohydrates; kcal, kilocalories; Pr, proteins; *, statistically significant. First line values represent Pearson’s partial correlation index, while the second line indicates *p*-values.

**Table 5:** The linear regression stepwise, age and sex adjusted.

Dependent Variables	Independent Variables	Beta	95% CL		*p*-Value
delta log non-HDL-C	log SFA T1	0.120	0.051	0.190	0.001 *
delta log LDL-C		0.112	0.033	0.191	0.006 *
delta log TC		0.092	0.033	0.150	0.002 *

Beta, linear regression coefficient adjusted for age and sex; CL, 95% confidence limits; *, statistically significant *p*-value ≤ 0.05.

## Data Availability

The records of patients are available in our local Informatic Database Trak-Care System.
